# Abdominal infection combined with pneumoperitoneum after renal transplantation: A case report

**DOI:** 10.1097/MD.0000000000032836

**Published:** 2023-02-03

**Authors:** Zhiming Deng, Huachen Zhu, Wei Du, Hongwei Zhang

**Affiliations:** a Organ Transplantation Department, The First People’s Hospital of Changde City, Changde, China.

**Keywords:** abdominal infection, pneumoperitoneum, renal transplantation

## Abstract

Abdominal infection combined with pneumoperitoneum after renal transplantation is rare, clinically confusing, and easily misdiagnosed by physicians as gastrointestinal perforation.

**Patient concerns:**

A 54-year-old man experienced abdominal pain and distension together with signs of peritoneal irritation after cadaveric renal transplantation. CT and standing abdominal plain film showed a large pneumoperitoneum in the abdominal cavity and the patient underwent an exploratory laparotomy but no gastrointestinal perforation was found.

**Diagnosis:**

No gastrointestinal perforation was found during the operation. In the search for the infectious agent, ascites culture was negative while next-generation sequencing was positive, suggesting the presence of intestinal flora ectopic to abdominal infection with anaerobic respiration fermentation leading to large amounts of gas.

**Interventions:**

The patient underwent exploratory laparotomy without gastrointestinal perforation, and then underwent abdominal lavage, placed abdominal drainage tube, and conducted culture and next-generation sequencing examination of ascites.

**Outcomes:**

Postoperative symptoms were relieved and intestinal function recovered. After 3 months of outpatient follow-up, the patient had stable transplanted kidney function and was in good spirits and sleeping well, with a good appetite, soft and regular stools, no abdominal pain and distension, and no fever.

**Conclusion:**

Patients after kidney transplantation should be wary of abdominal infection being misdiagnosed as gastrointestinal perforation.

## 1. Introduction

Severe intra-abdominal infections are common and significant problems in the intensive care unit, and the abdomen often ranks first among the sources of infection or sepsis. Peritonitis is generally classified as primary, secondary, or tertiary.^[1]^ However, there are few reports on pneumoperitoneum secondary to abdominal infection after renal transplantation, and easily misdiagnosed by physicians as gastrointestinal perforation. We describe a case of abdominal infection combined with pneumoperitoneum that occurred soon after renal transplantation.

## 2. Case report

A 54-year-old man was admitted to the hospital with the complaint of “maintenance hemodialysis for 7 years,” and was diagnosed with “renal end-stage renal disease,” with a previous history of “coronary atherosclerotic heart disease.” The patient underwent cadaveric donor allogeneic kidney transplantation, with the transplanted kidney placed in the left iliac fossa. He was then treated with ATG + glucocorticoid induction therapy, a triple prophylactic anti-rejection regimen of cyclosporine + Mycophenolate Mofetil + prednisone, as well as anti-infection therapy with meropenem. The urine output was insufficient in the early postoperative period, so hemodialysis was performed once on the third postoperative day, after which the patient’s urine output gradually increased and the blood creatinine level began to decrease.

**Figure 1.**
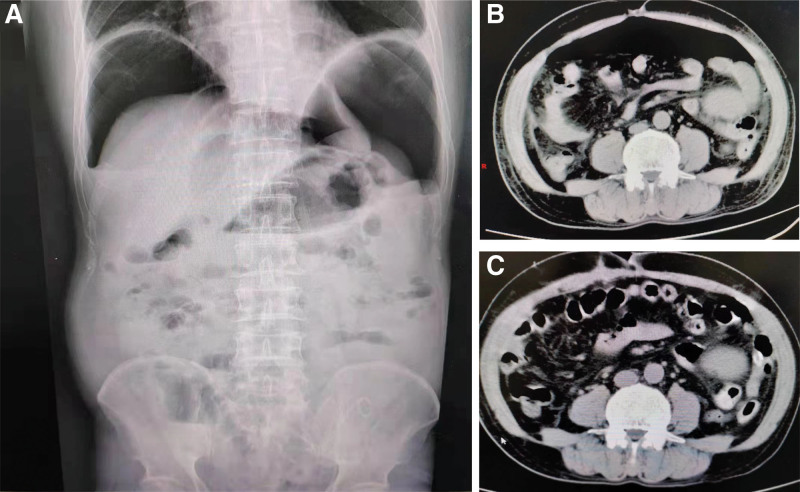
(A) The plain abdominal radiograph showed a crescent-shaped free gas shadow under diaphragms and scattered short liquid gas planes in the abdomen. (B) The abdominal CT showed a newly emerging pneumoperitoneum. (C) CT images after exploratory laparotomy. CT = computed tomography.

The patient had 1 black stool on the eighth postoperative day, although his blood pressure and hemoglobin remained stable. The following day, the patient developed abdominal pain and distension, and physical examination of the abdomen revealed both pressure and rebound pain, while the right lower abdomen was slightly obvious, without fever. The abdominal CT was repeated and revealed a newly emerging pneumoperitoneum (Fig. 1B), while the plain abdominal radiograph showed a crescent-shaped free gas shadow under diaphragms and scattered short liquid gas planes in the abdomen (Fig. 1A). After consulting the general surgery department, an exploratory laparotomy was performed.

### 2.1. Intraoperative findings

There was a large amount of gas in the abdominal cavity, as well as about 500 mL of yellow pus. Irregular inter-intestinal abscesses were visible in the right lower abdomen and pelvic cavity, with a large amount of pus moss attached to the surface of the intestinal canal, together with congestion and edema of the intestinal wall. Exploration showed no perforation of the stomach, duodenum, small intestine, colon, or rectum; the appendix was unevenly thickened, hard in texture, congested and edematous, and no perforation was observed. After joint consultation between the general surgery, organ transplantation, gastroenterology, and infection departments, it was unanimously agreed to remove the appendix. The abdominal cavity was flushed, and colonic paracolic drains and pelvic drains were placed for drainage.

**Figure 2.**
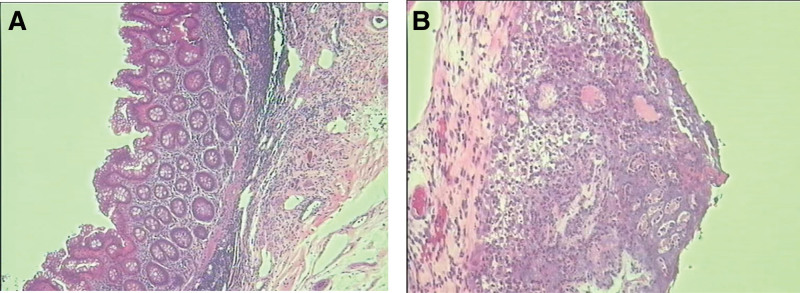
(A and B) Postoperative pathological analysis of the appendix showed chronic appendicitis with acute suppurative inflammation of the plasma membrane surface and fibrous tissue hyperplasia.

After the operation, the patient was transferred to the intensive care unit. The patient then developed fever, with elevated levels of urea nitrogen, serum creatinine, and serum potassium. He received blood purification treatment to remove metabolic waste and inflammatory factors. The oral anti-rejection drugs were discontinued at the same time, while methylprednisolone 40 mg/d was given intravenously together with imipenem-cilastatin anti-infection treatment and nutritional support. The anus was deflated on the second postoperative day. He was transferred back to the general ward 4 days after the operation, showed yellow stools, and received diet restoration and triple anti-rejection therapy. The patient’s temperature returned normal on the seventh postoperative day. The abdominal drainage tube was gradually removed on the eighth postoperative day, and repeat CT showed a significant reduction in the pneumoperitoneum compared with the preoperative pneumoperitoneum (Fig. 1C). Postoperative pathological analysis of the appendix showed chronic appendicitis with acute suppurative inflammation of the plasma membrane surface and fibrous tissue hyperplasia (Fig. 2A and B). The culture results of the intraoperative ascitic fluid were negative, while next-generation sequencing (NGS) detected the presence of *Proteus vulgaris*, *Bacteroides ovatus*, *Rumen bacteria*, *Enterococcus faecium*, and *Bifidobacterium longum*. After further treatment, the creatinine level finally decreased to 99 µmol/L by the time of discharge. As infection and dehiscence of the wound occurred after exploratory laparotomy, debridement and suturing were performed after dressing changes to control the infection, with the removal of sutures 4 weeks after surgery, at which time the wound had healed well. After 3 months of outpatient follow-up, the patient had stable transplanted kidney function and was in good spirits and sleeping well, with a good appetite, soft and regular stools, no abdominal pain and distension, and no fever.

## 3. Discussion

Patients’ bodies are adversely affected by end-stage renal disease and its complications, as well as postoperative immunosuppressive therapy, resulting in immunocompromise. Kidney transplantation recipients are at high risk of infection from pathogens including viruses, bacteria, and fungi, with sites of infection commonly found in the respiratory, urinary, and gastrointestinal tracts, as well as at the site of the surgical incision.^[2]^ The vast majority of kidney transplants do not enter the abdominal cavity, so abdominal infections rarely occur in kidney transplant recipients without peritoneal dialysis tubing. Abdominal infections are reported to occur most commonly after liver transplantation.^[3]^ The abdominal infection seen in this patient is a rare occurrence and was thus worth exploring as the abdominal cavity had not been invaded during the renal transplantation and he had no previous history of peritoneal dialysis.

The intestinal flora represents the largest micro-ecosystem in the body, with about 1014 bacteria residing in the intestine. These bacteria belong to 30 genera and 500 species and are mainly composed of anaerobic, facultative anaerobic, and aerobic bacteria, among which Lactobacillus and Bifidobacterium are the principal specialized anaerobic bacteria, together accounting for about 95% of the intestinal flora.^[4]^ The intestinal flora and the body are in a dynamic balance and maintain the health of the body; disruption of this balance is often manifested as a decrease in the proportion of beneficial bacteria, overgrowth of pathogenic bacteria, conversion of conditionally pathogenic bacteria into pathogenic bacteria, and promotion of disease progression. Beneficial bacteria in the intestinal tract enhance the barrier function of the gastrointestinal mucosa by adhering to the intestinal epithelium, forming a micro-ecological barrier to prevent colonization and invasion of the epithelium by pathogenic bacteria.^[5]^ In this case, carbapenem broad-spectrum antibiotics were started early postoperatively, which may have led to the killing of sensitive flora in the intestine and greatly reduced the number and diversity of probiotic bacteria, thus allowing colonization and proliferation of pathogenic bacteria, resulting in a microecological imbalance.^[6]^ NGS of the patient’s peritoneal fluid revealed that the infecting bacteria in the peritoneal cavity belonged to the normal human intestinal flora. It is likely that disruption of intestinal defense associated with the underlying disease and the use of high doses of corticosteroids and other immunosuppressive drugs during the perioperative period of transplantation, led to the presence of ectopic intestinal flora and abdominal infection.^[7]^ It is worth noting that NGS was able to detect the ectopic bacteria while the ascites culture was negative. NGS allows the simultaneous sequencing of thousands to billions of DNA fragments and can amplify and detect free DNA and DNA fragments in dead cells, making it possible to quickly identify pathogens in culture-negative specimens. This highlights the practical value of NGS in the detection of pathogens when traditional bacterial detection is ineffective.^[8]^ Culture negativity may also have been associated with the use of carbapenem antibiotics immediately after surgery. When anaerobic bacteria translocate from the intestine to the abdominal cavity and cause infection, they will produce large amounts of carbon dioxide gas through anaerobic respiratory fermentation,^[9]^ which explains the large amount of gas observed in the abdominal cavity by CT.

The patient presented with peritonitis after the onset of the disease, but without fever and shock, which may be attributed to the use of glucocorticoid. Cortisol has a clear inhibitory effect on pro-inflammatory mediators, and inhibits endothelial cells and neutrophil activation, thus blocking the acute phase response.^[10]^ The presence of right lower abdominal pressure rebound pain and pneumoperitoneum inclined the physician to suspect appendicitis and gastrointestinal perforation; however, the findings of both the surgical exploration and postoperative pathological analysis ruled out the above. Due to the high mortality rate in patients with the gastrointestinal perforation complication of peritonitis without aggressive surgery,^[11]^ even if the preoperative diagnosis is not completely precise, aggressive surgery is more likely to be the preference of general surgeons, while abdominal irrigation and drainage also play a positive role in controlling infection, albeit at the cost of surgical strikes and wound infection. In the last century, with the continuous development and maturation of laparoscopic techniques, laparoscopic exploration has been widely used for the early diagnosis and treatment of acute abdominal disease.^[12]^ In this case, the general surgeons were concerned about the effect of abdominal pressure on the transplanted kidney and the restriction of the transplanted kidney to the position of the trocar, together with other factors, and an open surgical plan was finally used. While successful cases of the laparoscopic management of abdominal disease in patients after renal transplantation have been reported, the number of cases is small,^[13]^ and the accumulation of successful experience has the potential to make laparoscopic surgery a preferred option for the management of abdominal disease in renal transplant recipients.

## Author contributions

**Conceptualization:** Hongwei Zhang.

**Data curation:** Huachen Zhu.

**Investigation:** Zhiming Deng, Huachen Zhu, Wei Du, Hongwei Zhang.

**Methodology:** Zhiming Deng.

**Validation:** Hongwei Zhang.

**Visualization:** Zhiming Deng, Wei Du.

**Writing – original draft:** Zhiming Deng.

**Writing – review & editing:** Hongwei Zhang.
